# The Heinous Hobnail: A Case Report of the Rare Hobnail Variant of Papillary Thyroid Carcinoma

**DOI:** 10.7759/cureus.61267

**Published:** 2024-05-28

**Authors:** MonishaRita Jayaraman, Lakshmipriya V, Volga Harikrishnan, Sridevi Manian

**Affiliations:** 1 Pathology, Saveetha Medical College and Hospital, Saveetha Institute of Medical and Technical Sciences, Saveetha University, Chennai, IND; 2 Pathology and Laboratory Medicine, Saveetha Medical College and Hospital, Saveetha Institute of Medical and Technical Sciences, Saveetha University, Chennai, IND

**Keywords:** thyroid pathology, overt hyperthyroidism, thyroidectomy, hobnail, aggressive variant of papillary carcinoma thyroid

## Abstract

The hobnail variant of papillary thyroid carcinoma (HVPTC) represents a distinctive and relatively rare histological subtype of thyroid malignancies. This variant is characterized by its unique cellular morphology with a hobnail appearance, that is, cells with apically positioned nuclei. There are other characteristics like micropapillary pattern and loss of cohesiveness of cells, which are indicative of HVPTC. It can be difficult to distinguish this pattern from other thyroid neoplasms; thus, a thorough microscopical examination is required. Thyroglobulin, thyroid transcription factor-1 (TTF-1), and other thyroid markers are commonly expressed by the tumor cells. Clinically, HVPTC is similar to conventional papillary thyroid cancer (PTC) in many aspects like incidence and epidemiology, but the former is associated with a worse prognosis. According to some research, the hobnail variety might behave more aggressively than conventional PTC, which highlights how crucial it is to identify and comprehend this distinct subtype. While the genetic and molecular underpinnings of HVPTC are still being elucidated, some studies have reported associations with specific genetic alterations, including BRAF, TP53, and TERT mutations. Investigating these molecular signatures may contribute to a better understanding of the variant's pathogenesis and potentially guide targeted therapeutic approaches in the future. In order to customize treatment plans, histopathology is essential in correctly diagnosing it. In this article, we present a case of PTC which presented as a solitary nodule on ultrasonogram in a 40-year-old female.

## Introduction

The most common malignancy of the endocrine organs is thyroid cancer. Papillary thyroid cancer (PTC) is the most common histological type of thyroid cancer, accounting for approximately 80-85% of all thyroid malignancies [1]. Its incidence has been steadily rising over the past few decades, with a particularly notable increase in recent years. This rise is believed to be multifactorial, attributed to improved diagnostic techniques, increased surveillance, and potentially environmental factors such as radiation exposure [1,2]. The incidence of PTC varies geographically, with higher rates observed in regions with higher iodine intake, such as parts of Asia. Additionally, there is a notable female predominance, with women being affected approximately three times more often than men. The peak incidence occurs in middle-aged individuals, typically between the ages of 30 and 50, although PTC can occur at any age [1-3]. PTC usually has a very good prognosis, whereas some subtypes of PTC are more aggressive in nature and have worse overall and disease-free survival rates [2,3]. The aggressive subtypes of PTC include tall cell, columnar, diffuse sclerosing, solid, and hobnail variants. Well-differentiated PTC is generally thought to be a rather indolent tumor with long-term survival rates >95% in the early stages [4]. The histology, cytology, molecular markers, therapeutic approaches, and results of these subtypes vary. This case, the hobnail variant of papillary thyroid carcinoma (HVPTC), accounts for 1% of all PTC [1]. They are associated with a more aggressive clinical course and poorer prognosis compared to classic PTC. One such case is discussed here in this article.

## Case presentation

**Figure 1:**
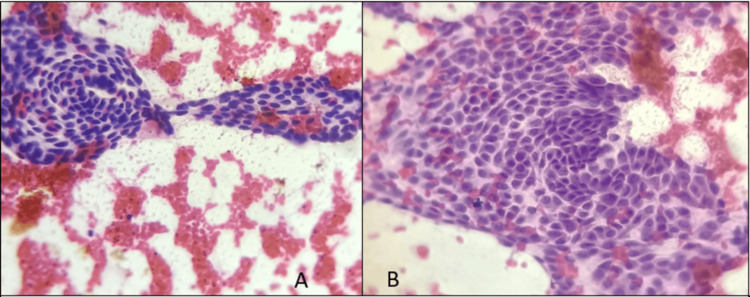
FNAC evaluation of the thyroid swelling (A) Cellular smears show micropapillary pattern without a central fibrovascular core in a hemorrhagic background (B) Higher magnification of a cluster shows teardrop or comet-like appearance of the cells due to tapering cytoplasm, with few cells showing mild nuclear atypia, atypical mitosis, and intranuclear inclusions FNAC: fine-needle aspiration cytology

A 40-year-old woman came to the outpatient department with complaints of weight gain, tremors, and dyspepsia. The patient also had complaints of palpitations and breathlessness, both of which were worsened on lying down. She has been on regular medication for hyperthyroidism for the past seven years. She underwent an ultrasonogram of the neck which revealed a fairly defined heteroechoic, predominantly hypoechoic lesion in the left lobe of the thyroid of size 2.6x2.4 cm with internal calcification and vascularity present. It was reported to be Thyroid Imaging Reporting and Data System (TI-RADS) 4, and fine-needle aspiration cytology (FNAC) was suggested. Ultrasound-guided FNAC of the lesion was done which showed sheets and papillary pattern of follicular epithelial cells with fibrovascular core. Cells showed nuclear crowding, overlapping, nuclear grooving, intranuclear inclusions, powdery chromatin, anatomical bordering, and streaming with scanty colloid in a hemorrhagic background (Figure 1). According to the 2023 Bethesda System for Reporting Thyroid Cytopathology, category VI malignant-papillary thyroid carcinoma was given.

**Figure 2:**
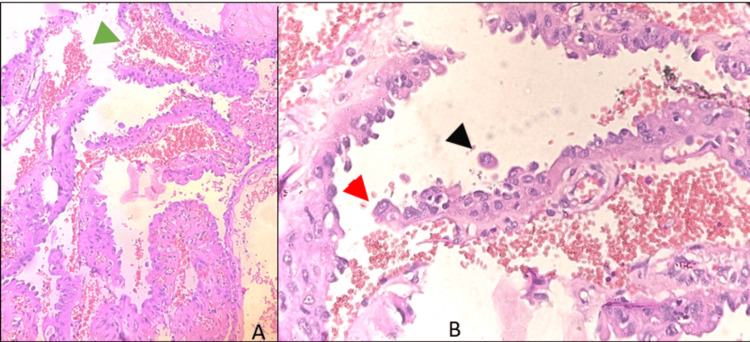
Histopathological examination of the thyroidectomy specimen (A) Section shows numerous papillary projections with central fibrovascular core (green arrow) (B) Higher magnification of the image shows discohesive cells (black arrow) with enlarged, apically placed nucleus protruding from the apical surface which is characteristic of hobnailing (red arrow)

The patient underwent total thyroidectomy following which we received in the histopathology lab a total thyroidectomy specimen measuring 9.5x5.5x4 cm. A solitary lesion was found in the left lobe measuring 2.5x1.5 cm. Under microscopy, sections showed a thyroid gland with a malignant neoplasm composed of cells arranged in papillae with fibrovascular cores and micropapillae lacking fibrovascular cores. Cells were pleomorphic showing nuclear clearing, and few had vesicular nuclei, nuclear grooving, intranuclear inclusions, and moderate eosinophilic cytoplasm. Areas with marked loss of cohesion of cells were seen, and the cells were large with an increased nucleus-to-cytoplasm ratio, apically placed protruding hyperchromatic, moderately pleomorphic nuclei, and dense eosinophilic cytoplasm. Few mitotic figures and areas of hemorrhage were appreciated (Figure 2). The hobnail areas were seen in more than 40% of the tumor area. In this case, hobnailing and micropapillary architecture were appreciated in almost 40% of the tumor, and thus, a diagnosis of HVPTC was made. The cells are positive for TTF-1 and thyroglobulin, similar to the conventional PTC. Similar to other PTC subtypes, this tumor shows strong cytoplasmic and membranous staining for cytokeratin.

## Discussion

The HVPTC was included in the WHO classification only in 2017, but it was first described much earlier [1]. Aisoli et al. in 2010 first described the HVPTC as a moderately differentiated PTC with poor prognosis. They described it as a lesion with discohesive areas showing cells with loss of polarity, increased nuclear-cytoplasmic ratio, and apically placed nucleus. Initially, HVPTC was thought to be areas of poor differentiation, but was later described as a clinical variant which is associated with a poor prognosis due to higher rates of mortality. Mortality in HVPTC was attributed to older age, increased incidence of lymph node metastases, extrathyroidal extension, distant metastases, faster spread, and refractoriness to radioiodine [2,3].

According to the recent WHO guidelines, the tumor must contain at least 30% of cells with hobnail-micropapillary characteristics in order to be classified as an HVPTC [4,5]. Although, if the hobnail component is less than the cutoff, this is 5-30%, it is still essential to identify and mention it in the final report of the patient as it is associated with adverse prognostic significance [5-7]. Several studies show that tumors with ≥30% hobnail morphology had more chances for developing lymph node metastases compared to those with <30% hobnail morphology [7]. Rates of distant metastases and high-risk pathological characteristics such as necrosis, extrathyroidal extension, and lymphovascular invasion did not differ significantly. BRAF mutations were seen in both groups [7].

HVPTC often has features of necrosis, atypical mitoses, and invasion into extrathyroidal tissues, blood vessels, and lymphatics. They are also associated with recurrence, local spread, and distant metastasis and thus are considered aggressive tumors. The percentage of hobnail characteristics in PTC was formerly thought to play a substantial effect in mortality. The 30% cut-off was suggested as a prognostic factor in a few of the studies investigating this aggressive type of PTC, and it was discovered that tumors exhibiting hobnail features in more than 30% of the tumor areas were associated with greater mortality [8]. A subsequent investigation using the same cut-off failed to reveal any variations in lymph node involvement, distant metastases, or mortality [9]. A prior meta-analysis involving 124 HVPTC patients indicated that patients with more than 30% hobnail cells had a higher rate of lymph node metastasis; however, no significant differences were observed in the rates of distant metastases or other clinicopathological features known to contribute to mortality [6]. There are conflicting results from different studies on the rate of metastasis and prognosis, and further studies should be encouraged to study in detail about HVPTC.

Many studies show that HVPTC is most commonly associated with BRAF V600E mutations followed by TP53 mutations in a lesser fraction of these tumors [5-7]. Studies showed there are an increased risk of local recurrences and a 5.3-fold higher rate of recurrence in the tumors which have 30% or more BRAF V600E allele [5,6]. The risk of distant spread is also increased with the lung and bone being the most frequent sites of metastases [7]. Since BRAF mutational genotypes have been associated with higher rates of disease recurrence, decreased disease-free survival, and decreased overall survival, this highlights the increased risk of cancer-related death in HVPTC patients [10,11]. HVPTC shows refractoriness to conventional treatments like radioactive iodine (RAI) [11]. Promising outcomes have been observed in BRAF-mutated PTC treated with targeted treatments, such as BRAF-specific inhibitors like dabrafenib and vemurafenib [11].

## Conclusions

HVPTC represents a distinctive entity within the realm of thyroid pathology. A thorough histological examination is needed for its identification. Immunohistochemistry is helpful to differentiate it from metastatic cancer from elsewhere or undifferentiated carcinoma. From a clinical perspective, comprehending the distinct characteristics of HVPTC is essential for proper patient care and highlights the continuous improvement of thyroid cancer categorization in the pathology domain.
